# High precision computer-generated moiré profilometry

**DOI:** 10.1038/s41598-019-44186-3

**Published:** 2019-05-24

**Authors:** Chengmeng Li, Yiping Cao, Lu Wang, Yingying Wan, Guangkai Fu, Yapin Wang, Cheng Chen

**Affiliations:** Department of Opto-Electronics, Sichuan University, Chengdu, 610064 China

**Keywords:** Physics, Optical physics

## Abstract

Recently, a computer-generated moiré profilometry was proposed by our research group. It can effectively avoid the influence of the transient caused by moiré fringes’ direct acquisition and generally owns a higher accuracy. But when the spatial spectrum of the captured deformed pattern is severely aliased caused by the measured object, the accuracy of this method may be affected to some extent due to the impure background light component extraction. So, a high precision computer-generated moiré profilometry based on background light component’s accurate elimination is proposed. By adding an additional special phase-shifting sinusoidal grating to accurately extract valid information in the spatial domain and improve the sinusoidal feature of the pattern, the measurement precision can be improved effectively. Though the single-shot feature is broken, the real-time measuring feature is still maintained successfully. Experimental results show the feasibility and validity of the proposed method.

## Introduction

Structured-lighting methods^1–3^ are important parts of the optical three-dimensional (3D) measurement. Fourier Transform Profilometry (FTP)^4–8^ and Phase Measurement Profilometry (PMP)^9–12^ are the prominent representative methods among them. FTP has firstly proposed by Takeda and can achieve phase retrieval using only one deformed pattern. But its measurement accuracy is somehow limited by the filtering operation in the frequency domain. While PMP shows a higher measurement accuracy for its multiple (>2) phase-shifting sinusoidal patterns’ projection and usually plays an important role in static measurement. There are many real-time measuring methods^13–18^ based on PMP, however, their accuracy limited by various factors is still an unsolved problem. Besides, Moiré Surface Profilometry^19–23^ with the merits of being stable and economical, is also a promising method in various measurements. In moiré-based surface measurements, the object surface information is recorded in the moiré fringes and the equal-depth contours can be used to determine the height distribution of the object. There are three major types of moiré surface profilometry. The first one is shadow moiré profilometry, in which moiré fringes are obtained by capturing the deformed pattern viewing through the projection grating at an angle. The second one is projection moiré profilometry, which requires a certain angle between two gratings to generate moiré fringes by capturing the projected grating viewing through an extra grating. In the last one, digital moiré profilometry, a computer-generated grating is projected onto the object and then captured by a camera, and the moiré fringes are generated by superimposing a grating of the same frequency over the captured deformed pattern afterwards. Lately, F. Mohammadi *et al*. proposed a single-frame digital phase-shifting moiré 3D shape measurement method^24,25^. In their approach, a synthetically computer-generated grid of the same frequency as the captured fringe pattern is overlaid on this fringe pattern and digitally shifted to generate multiple phase-shifted patterns in the post-process. Since phase-shifting is performed digitally rather than optically, this proposed method avoids complicated setup, and permits flexibility in adjusting both the grid period and phase shift in comparison with traditional moiré profilometry (shadow moiré and projection moiré). But a single-frame grid removal technique is necessary to remove both straight and curved grid lines, which demands complex digital processing.

Recently, our group proposed a computer-generated moiré profilometry (CGMP)^26^ which captures the deformed patterns rather than moiré fringes themselves to achieve phase retrieval. It effectively avoids the influence of the transient caused by moiré fringes’ direct acquisition and generally owns a higher accuracy. But when the spatial spectrum of the captured deformed pattern is severely aliased, its accuracy may be affected to some extent due to the impure background light component elimination. In order to solve this problem and improve the measurement accuracy, there is another idea occurs for eliminating the background light by adding another 180-degree phase-shifted grating to extract valid information in the spatial domain instead of the frequency domain. It is defined as high precision computer-generated moiré profilometry (HCGMP), which generally owns the advantages of restraining high frequency noise and improving the sinusoidal feature of the fringe patterns. Furthermore, the real-time measuring feature of HCGMP is still maintained though the single-shot feature is substituted by the two-frame feature.

## Methods

**Figure 1:**
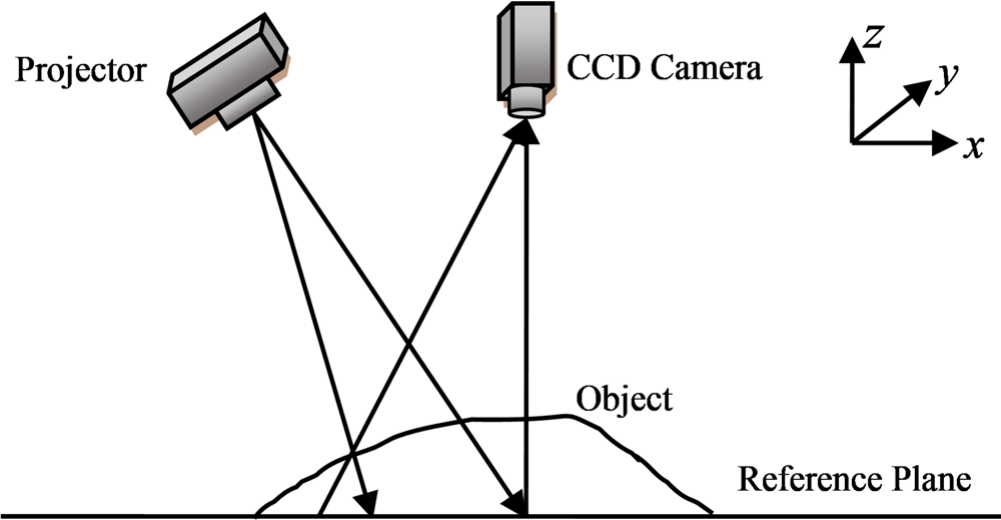
Optical setup of the proposed method.

The optical setup of the proposed method mainly consists of a DLP projector and a CCD camera, and its schematic diagram is shown in Fig. 1. This method consists of two steps: preparation and measurement.

### Preparation

As described in the previously proposed computer-generated moiré profilometry^26^, the previous preparation must be accomplished. According to its principle, when four sinusoidal gratings with a phase difference of 90-degree in turn are projected onto the reference plane respectively, four fringe patterns captured from the reference plane can be expressed as Eqs (1–4):

1 $${I}_{R1}(x,y)=R(x,y)\{a+b\,\cos [2\pi fx+{\phi }_{0}(x,y)]\},$$

2 $${I}_{R2}(x,y)=R(x,y)\{a+b\,\cos [2\pi fx+{\phi }_{0}(x,y)+\pi /2]\},$$

3 $${I}_{R3}(x,y)=R(x,y)\{a+b\,\cos [2\pi fx+{\phi }_{0}(x,y)+\pi ]\},$$

4 $${I}_{R4}(x,y)=R(x,y)\{a+b\,\cos [2\pi fx+{\phi }_{0}(x,y)+{\rm{3}}\pi /2]\},$$

where *R*(*x, y*) presents the reflectivity coefficient of the reference plane, *a* donates the background component and *b* reflects the fringe contrast, *f* is the frequency of the fringe pattern, *φ*_0_(*x, y*) is the phase distribution modulated by the reference plane. Due to the 180-degree phase difference between *I*_*R*1_ and *I*_*R*3_, *I*_*R*2_ and *I*_*R*4_, two alternating components (AC) on reference plane with 90-dergree phase difference can be refined extracted by Eqs (5 and 6).

5 $${I}_{R1}^{AC}(x,y)=R(x,y)b\,\cos [2\pi fx+{\phi }_{0}(x,y)]=\frac{1}{2}[{I}_{R1}(x,y)-{I}_{R3}(x,y)],$$

6 $${I}_{R2}^{AC}(x,y)=R(x,y)b\,\cos [2\pi fx+\pi /2+{\phi }_{0}(x,y)]=\frac{1}{2}[{I}_{R2}(x,y)-{I}_{R4}(x,y)].$$

Then, these two ACs need to be stored in computer in advance.

### Measurement

**Figure 2:**
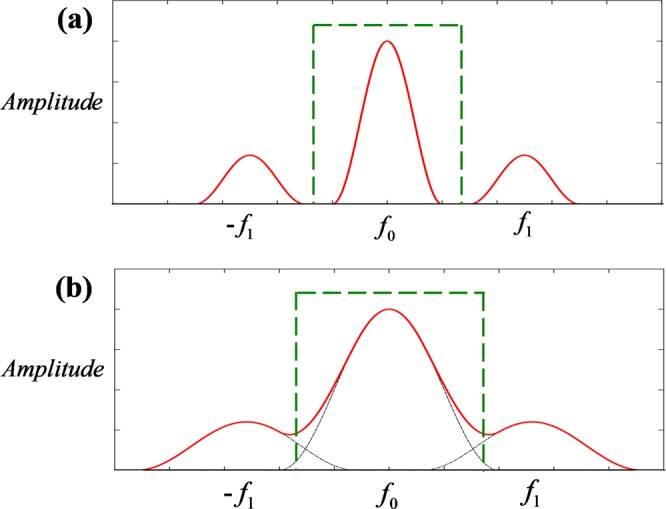
The diagram of frequency spectrum: (**a**) without aliasing; (**b**) under aliasing.

While measuring, the AC of the deformed pattern is also required to be extracted and its purity directly affects the measuring accuracy. As shown in Fig. 2(a), when the spectrum of the deformed pattern is discrete, just by using a low-pass filter can the direct component (DC) separated out. It means that the AC of the deformed pattern can be purely extracted. Thus, the measuring accuracy can be guaranteed. But if the AC overlaps with the DC in the spectrum, spectrum aliasing occurs at this condition, the above filtering method may be limited to some extent. The more serious spectrum aliasing is, the lower accuracy will be. Just as shown in Fig. 2(b), DC is severely aliased with AC, where the solid line represents the actual distribution of the spectrum and the dotted line indicates the distribution of DC and AC ought to be. Obviously, the filtering operation is unable to extract AC purely, and the measuring accuracy might not be guaranteed. In order to improve the measuring accuracy even if the spectrum aliasing occurs, we propose a new method which adding another grating with 180-degree phase difference to purely extract AC in the spatial domain instead of the frequency domain.

The two deformed patterns modulated by the measured object can be expressed as Eqs (7 and 8):

7 $${I}_{o1}(x,y)={R}^{\text{'}}(x,y)\{a+b\,\cos [2\pi fx+\phi (x,y)]\},$$

8 $${I}_{o2}(x,y)={R}^{\text{'}}(x,y)\{a+b\,\cos [2\pi fx+\pi +\phi (x,y)]\},$$

where ${R}^{\text{'}}(x,y)$ is the reflectivity coefficient of the object surface, and *φ*(*x, y*) is the phase distribution modulated by the object surface. Due to the 180-degree phase difference between these two deformed patterns, half of the two patterns’ subtraction, as shown in Eq. (9), is the AC (${I}_{o1}^{AC}(x,y)$) of the first deformed pattern.

9 $${I}_{o1}^{AC}(x,y)={R}^{\text{'}}(x,y)b\,\cos [2\pi fx+\phi (x,y)]=\frac{1}{2}[{I}_{o1}(x,y)-{I}_{o2}(x,y)].$$

When this remained AC of the deformed pattern multiplied with those of the fringe patterns, the products can be expressed as Eqs (10–11).

10 $$\begin{array}{rcl}{I}_{1}^{\ast }(x,y) & = & {I}_{o1}^{AC}(x,y)\times {I}_{R1}^{AC}(x,y)\\  & = & \frac{1}{2}A(x,y)\cos [4\pi fx+\phi (x,y)+{\phi }_{0}(x,y)]\\  &  & +\,\frac{1}{2}A(x,y)\cos [\phi (x,y)-{\phi }_{0}(x,y)],\end{array}$$

11 $$\begin{array}{rcl}{I}_{2}^{\ast }(x,y) & = & {I}_{o1}^{AC}(x,y)\times {I}_{R2}^{AC}(x,y)\\  & = & -\frac{1}{2}A(x,y)\sin [4\pi fx+\phi (x,y)+{\phi }_{0}(x,y)]\\  &  & +\,\frac{1}{2}A(x,y)\sin [\phi (x,y)-{\phi }_{0}(x,y)].\end{array}$$

Where *A*(*x, y*) denotes $R(x,y){R}^{\text{'}}(x,y){b}^{2}$ to simplify the equations. The last terms in Eqs (10 and 11) are exactly the cosine and sine of the phase only caused by the object. They can be easily extracted by a low-pass filter, which are defined as 0-degree moiré fringe as shown in Eq. (12) and 90-degree moiré fringe as shown in Eq. (13).

12 $${I}_{moire0}=\frac{1}{2}A(x,y)\cos [\phi (x,y)-{\phi }_{0}(x,y)],$$

13 $${I}_{moire90}=\frac{1}{2}A(x,y)\sin [\phi (x,y)-{\phi }_{0}(x,y)].$$

Obviously, the fringe contrast coefficient and the reflectivity of the reference plane and object are removed when dividing Eq. (13) by Eq. (12) to get the tangent of the phase only caused by the object.

14 $$\phi (x,y)-{\phi }_{0}(x,y)=\arctan (\frac{{I}_{moire90}}{{I}_{moire0}}).$$

The phase only caused by the object in Eq. (14) is wrapped between [−*π*, *π*) due to arctangent operation. Therefore, phase unwrapping operation is required to transform it into unwrapped phase *ϕ*(*x, y*), and the 3D surface, expressed as *h*(*x, y*), can be reconstructed successfully according to the phase-to-height mapping relation^27^ shown in Eq. (15).

15 $$\frac{1}{h(x,y)}=a(x,y)+b(x,y)\frac{1}{\varphi (x,y)}+c(x,y)\frac{1}{{\varphi }^{2}(x,y)}.$$

## Results and Discussions

**Figure 3:**
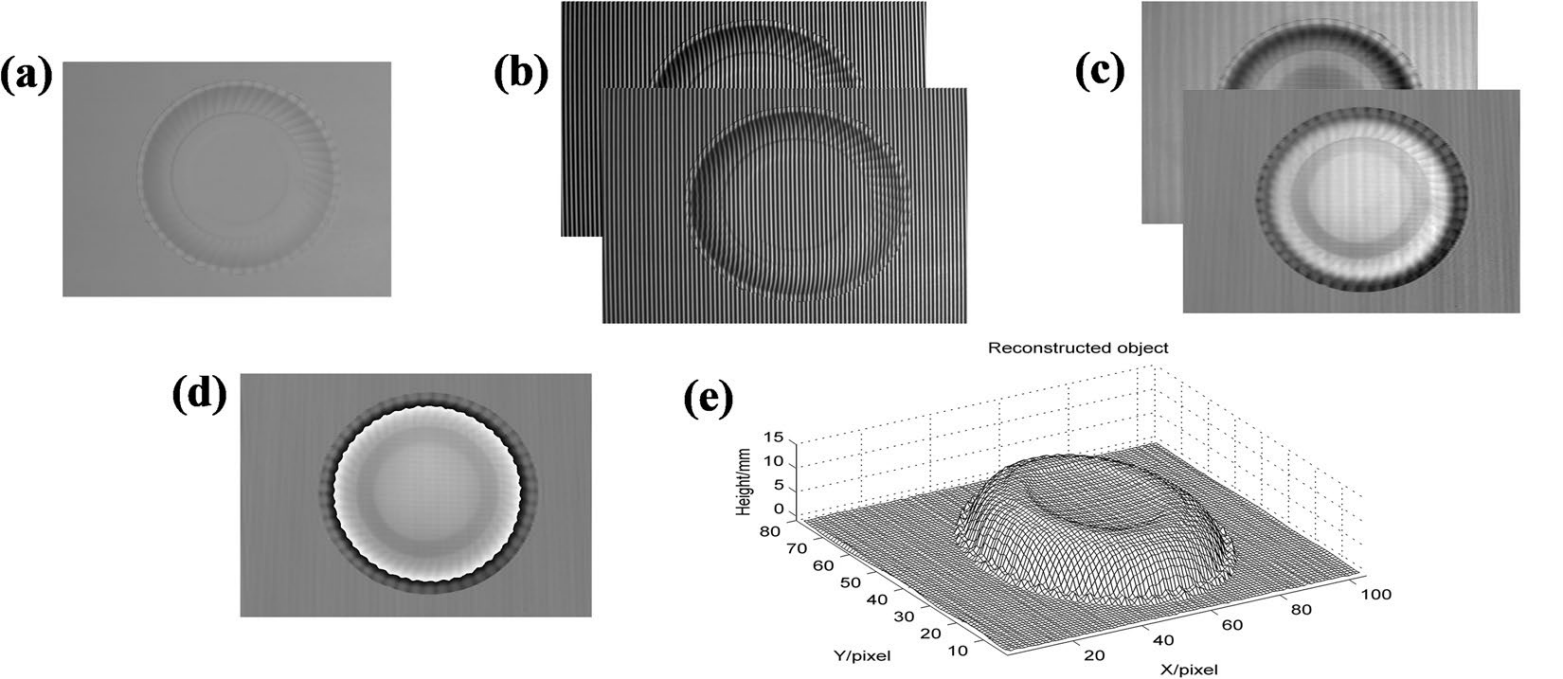
Experimental results of a platter based on HCGMP: (**a**) the measured object; (**b**) two deformed patterns; (**c**) two moiré fringes; (**d**) the wrapped phase; (**e**) the reconstructed object.

The experimental system is built as Fig. 1, where the DLP is View Sonic PLED-W200 and the CCD camera is MVC1000MF. A platter as shown in Fig. 3(a) is measured. When measuring, the two sinusoidal gratings are on purpose projected onto the object respectively and the corresponding captured deformed patterns are shown in Fig. 3(b). Two moiré fringes generated by the proposed method are shown in Fig. 3(c), whose ratio is just the tangent of the phase only caused by the object. Then, the wrapped phase is shown in Fig. 3(d). Finally, the reconstructed object (Fig. 3(e)) can be retrieved successfully by phase-to-height mapping. The experimental results show that the proposed method is feasible and valid.

**Figure 4:**
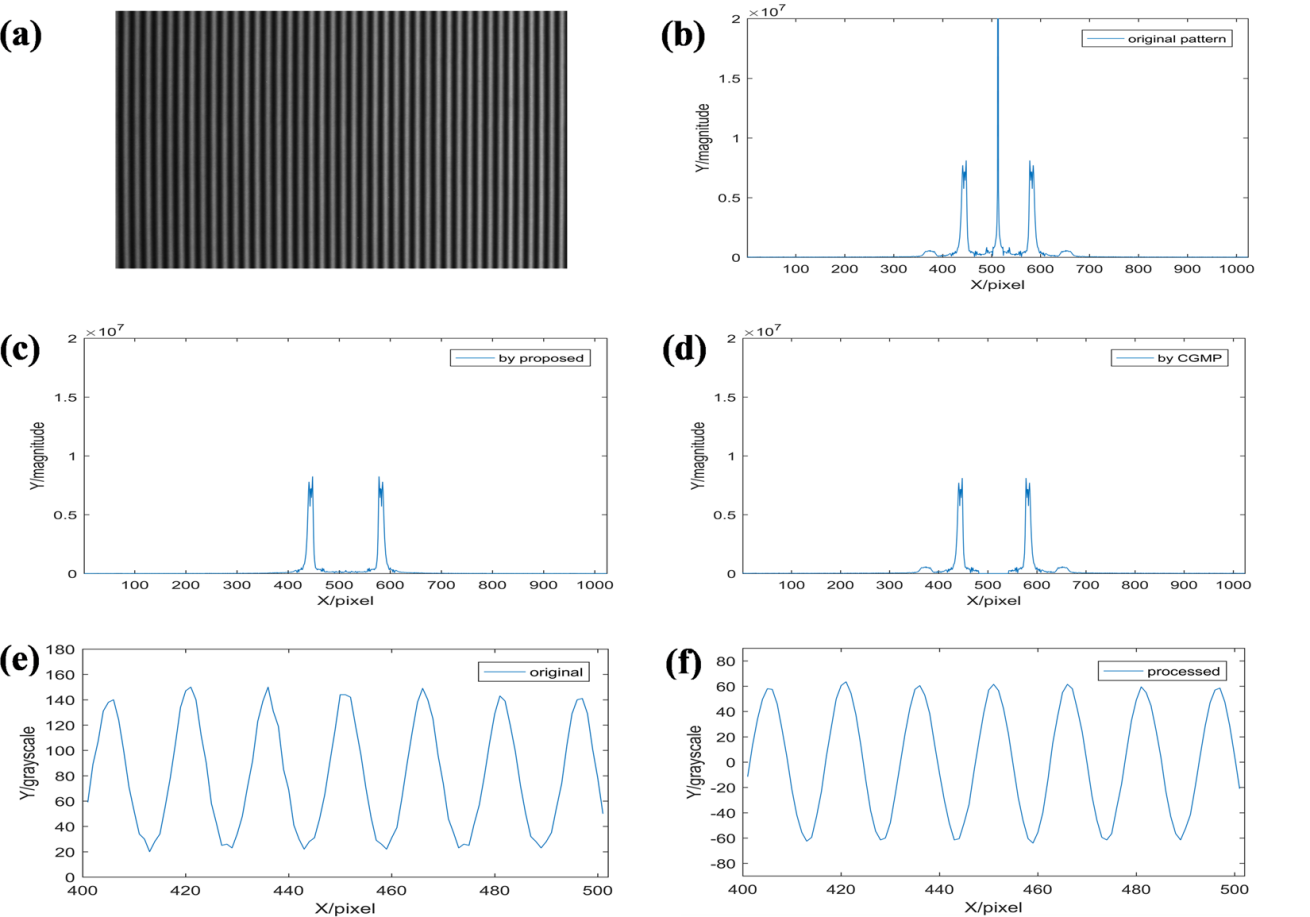
The spectrum analysis: (**a**) the original fringe pattern; (**b**) the frequency spectrum of (**a**); (**c**) the fundamental frequency component obtained by HCGMP; (**d**) the fundamental frequency component obtained by CGMP; (**e**) the cutaway view of (**a**) in the 400–500 pixel; (**f**) the corresponding cutaway view of the fringe pattern after background light elimination by HCGMP.

Compared with CGMP, HCGMP has the merits in background light component elimination and high frequency noise restriction. The spectrum analysis of the fringe patterns (Fig. 4) indicates the effective elimination of the background light and high frequency noise by our proposed method.

In the HCGMP, an extra grating with 180-degree phase difference from the original grating needs to be projected and the corresponding deformed pattern must be captured. So, the background light component can be exactly eliminated by subtracting the captured extra fringe pattern from the original fringe pattern. As can be seen in Fig. 4(a), it is the captured original fringe pattern from the reference plane. The frequency spectrum of this original fringe pattern is shown in Fig. 4(b). And the frequency spectrum of the fundamental frequency component obtained by HCGMP is shown in Fig. 4(c), when compared with the spectrum obtained by CGMP as shown in Fig. 4(d), it is clearly that the zero frequency, spectrum of the background light, is eliminated more precisely. Simultaneously, some high frequency noise is also removed. For a better observation, the cutaway view of partial original fringe pattern is shown in Fig. 4(e), its grayscale range is approximate between 20 and 140. It is easy to see that its sinusoidal feature is a little poor. The fringe pattern after background light component eliminated by HCGMP is shown in Fig. 4(f), its grayscale range is moved to −60 to 60. Meanwhile, the sinusoidal feature of the pattern is improved efficiently, and the random noise is restrained simultaneously.

In order to quantitatively test the precision of HCGMP, several height-known flat planes are measured respectively, and three parameters are used to describe the measuring performance. They are average height ($\overline{h}$), mean absolute error (MAE) and root mean square error (RMS) to express the mean height of the plane, the measurement accuracy and the measurement repeatability respectively. The experimental results obtained by CGMP, 4-step PMP and the HCGMP are shown in Table 1. Compared to CGMP, HCGMP has the higher accuracy and nearly equivalent to 4-step PMP.

**Table 1:** Experimental results for known heights by different methods (/mm).

*h*	10			14			23		
Method	CGMP	4-PMP	HCGMP	CGMP	4-PMP	HCGMP	CGMP	4-PMP	HCGMP
$\bar{h}$	10.030	9.974	10.020	14.006	13.990	13.990	23.021	23.977	23.019
MAE	0.103	0.082	0.080	0.105	0.091	0.089	0.126	0.102	0.111
RMS	0.136	0.097	0.102	0.138	0.112	0.112	0.158	0.125	0.137

**Figure 5:**
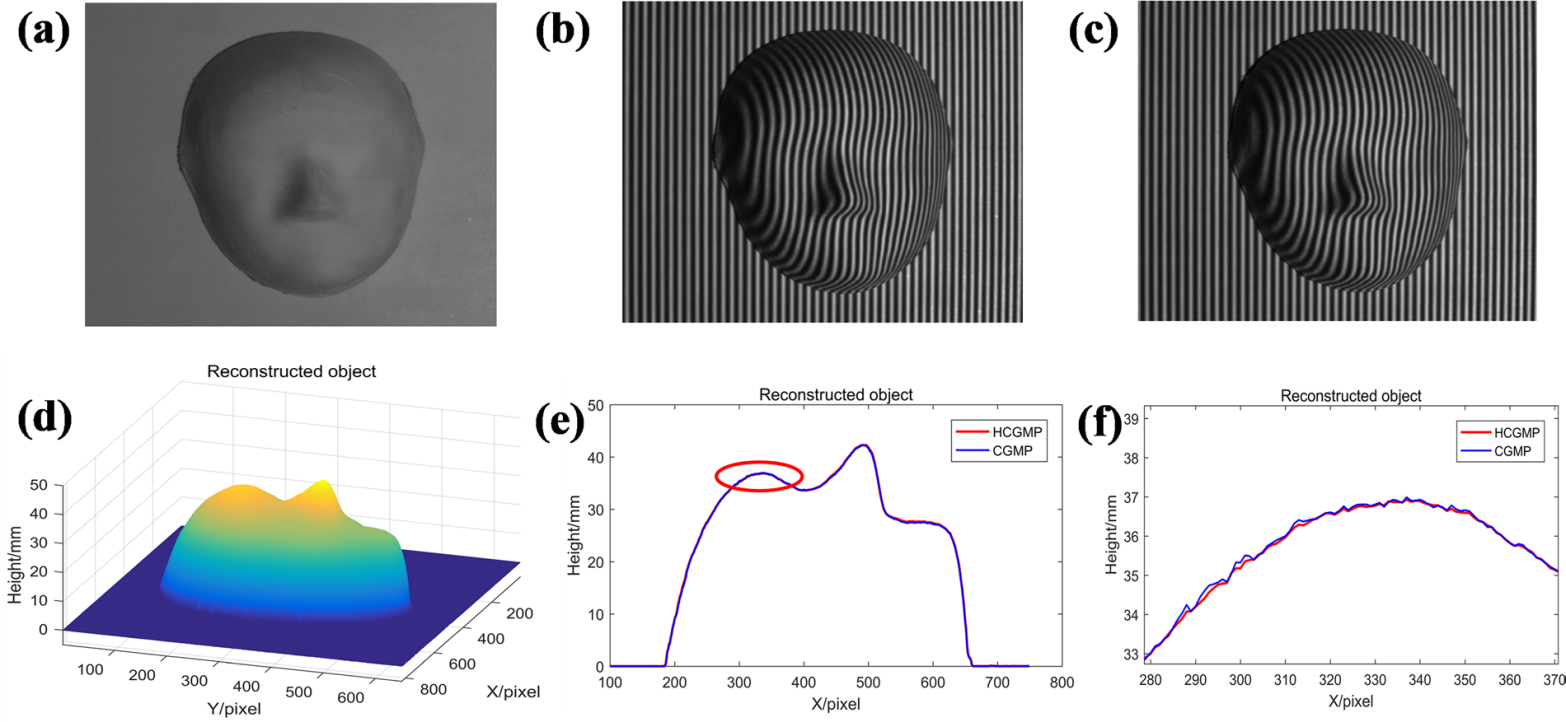
The accuracy comparison between CGMP and HCGMP: (**a**) the measured face mask; (**b**) the first deformed pattern; (**c**) the second deformed pattern; (**d**) the reconstructed object by HCGMP; (**e**) the cutaway view at column 650 of the results by CGMP and HCGMP; (**f**) the enlarged view in the ellipse of the (**e**).

Furthermore, we take experiments using HCGMP and CGMP respectively, and the results are shown in Fig. 5. We use a face mask (Fig. 5(a)) as the original object to be measured. And the two captured complementary deformed patterns are shown in Fig. 5(b,c). Finally, the reconstructed result (Fig. 5(d)) can be obtained successfully by HCGMP. According to the diagram, it completely reconstructs the 3D shape of the face mask, and the surface is fairly smooth. For comparison, the CGMP is also used to reconstruct the same object. The cutaway views at the same 650^th^ column extracted from the results by CGMP and HCGMP respectively are shown in Fig. 5(e). In order to better observe the difference between two measuring results, the mask forehead circled by an ellipse in Fig. 5(e) is enlarged and shown in Fig. 5(f). It is quite clear that HCGMP indicates an effective reconstruction result when measure a simple object since its result is smooth while that by the CGMP has lots of burrs.

**Figure 6:**
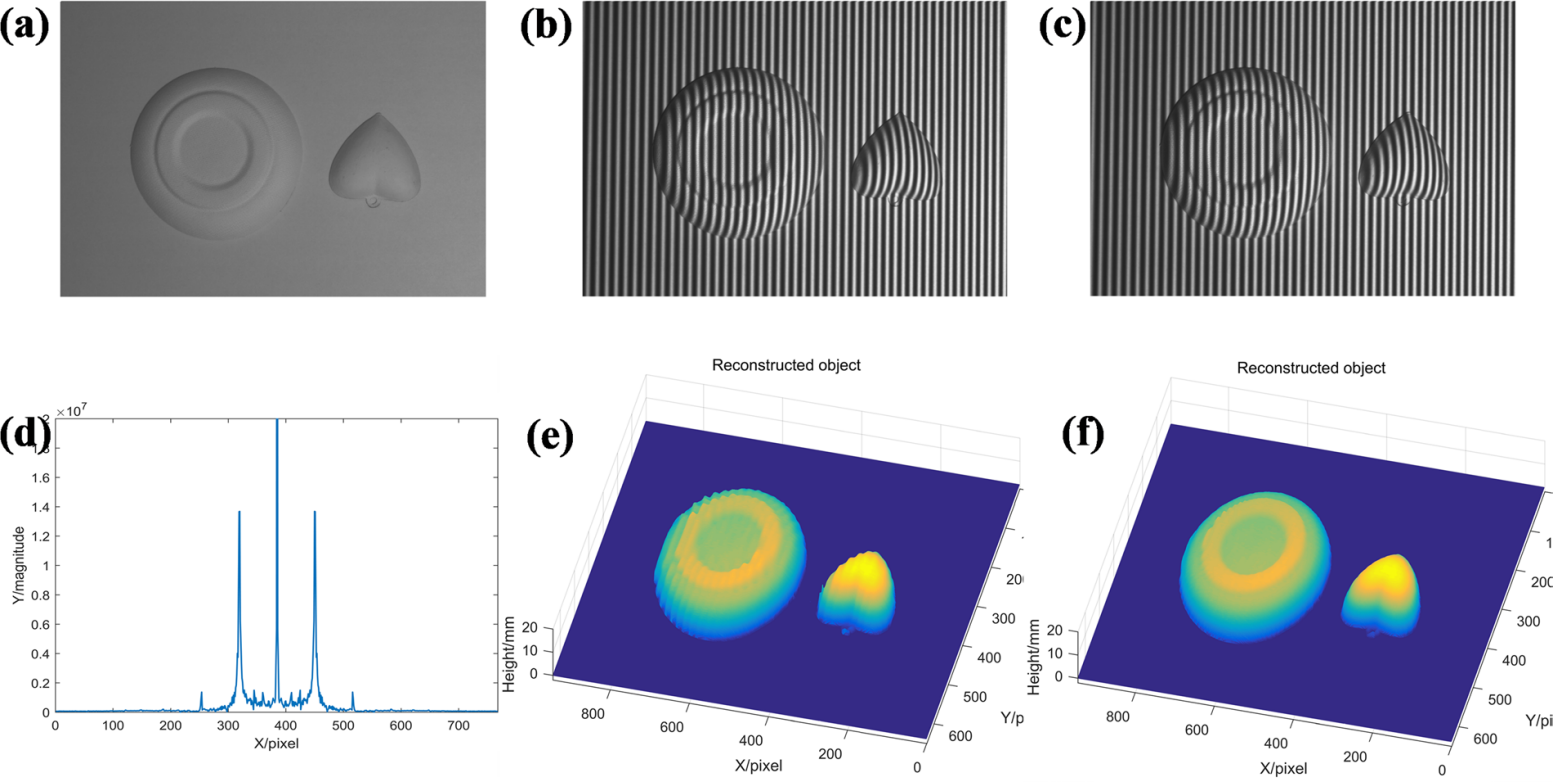
Comparison between CGMP and HCGMP: (**a**) the measured object; (**b**) the first captured deformed pattern; (**c**) the second captured deformed pattern; (**d**) the spectrum of the first deformed pattern; (**e**) the reconstructed object by CGMP; (**f**) the reconstructed object by HCGMP.

Further to achieve an intuitive demonstration of HCGMP’s effective measuring capacity in the case of spectrum aliasing, the experimental results respectively by CGMP and HCGMP are accomplished as shown in Fig. 6. The fundamental frequency of projected sinusoidal grating is purposively designed smaller and the measured object is set to be more complex to let the fundamental frequency spectrum caused by measured object definitely aliased with the zero frequency spectrum in each captured deformed pattern.

A composited object with an isolated heart model and an isolated platter as shown in Fig. 6(a) is measured and two captured complementary deformed patterns are shown in Fig. 6(b,c) respectively. Figure 6(d) is the spectrum of the deformed pattern in Fig. 6(b). It reveals that the fundamental frequency spectrum caused by the composited object is definitely aliased with the zero frequency spectrum. The reconstructed object by CGMP is shown in Fig. 6(e) while that by HCGMP is shown in Fig. 6(f). Obviously, some undesirable textures appear on the surface of the reconstructed object by CGMP, because of the spectrum aliasing, while the reconstructed 3D shape by HCGMP is smooth and shows a high fidelity. It shows that HCGMP keeps the higher accuracy even though the spectrum aliasing exists.

In addition to improving the measurement accuracy, this method can also realize real-time measurements. Since two fringes are required to reconstruct the object, it is more concise for real-time measuring than PMP. By means of projecting two gratings in a high-speed circularly and capturing the deformed patterns synchronously, real-time measurement can be achieved.

**Figure 7:**
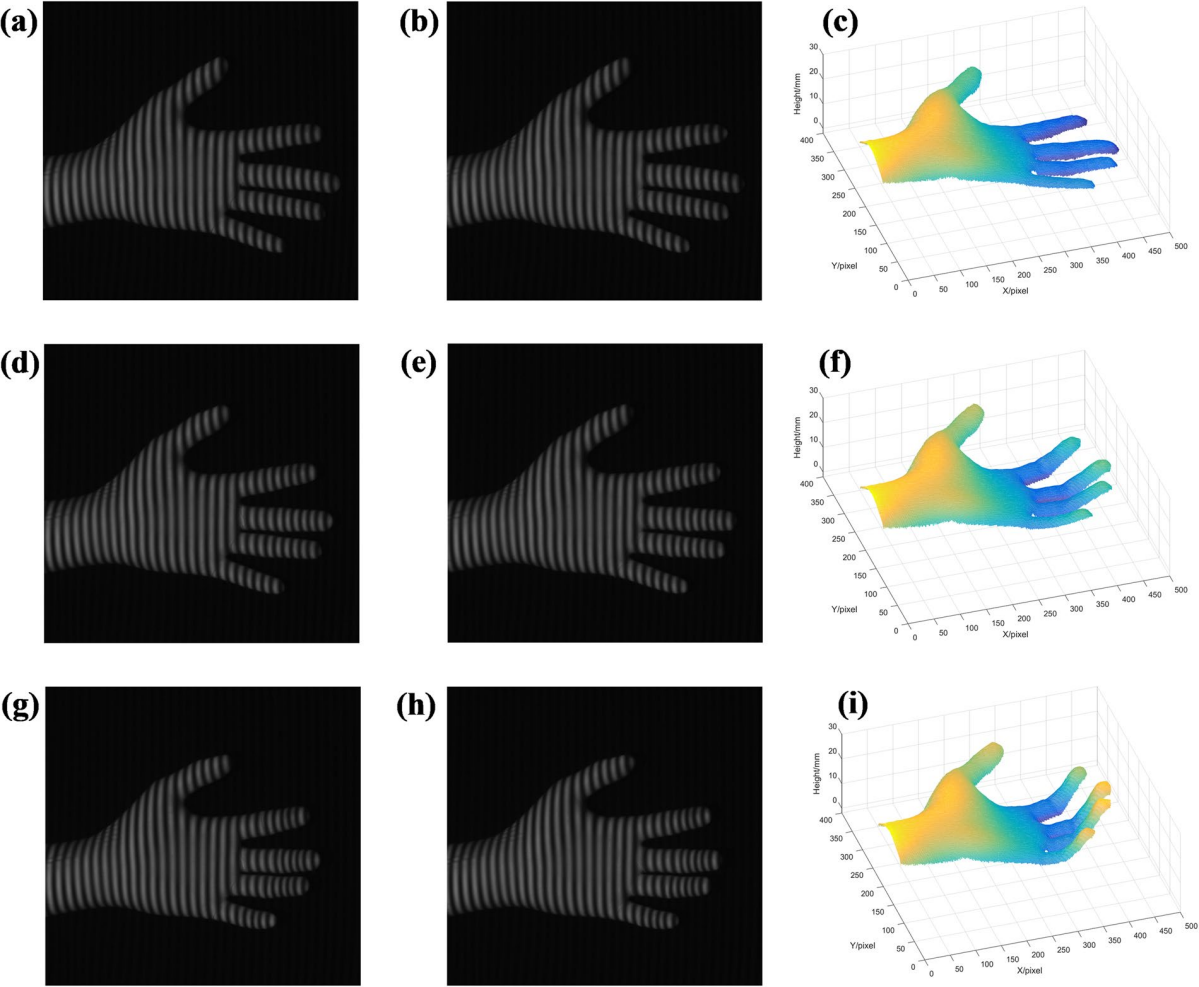
Real-time measuring result: (**a,b**) the two deformed patterns at status 1; (**c**) the reconstructed object at status 1; (**d**,**e**) the two deformed patterns at status 2; (**f**) the reconstructed object at status 2; (**g**,**h**) the two deformed patterns at status 3; (**i**) the reconstructed object at status 3.

Here, we used a DLP projector which can be controlled by a designed timing sequence to refresh 8-bit image up to 120 frames per second (fps). A high frame rate CCD camera can capture the deformed patterns synchronously by the synchronous signal. The reconstruction rate of this method can reach up to 60 fps. A palm in motion is measured. Due to the large amount of measurement data, we randomly selected the experimental results at three different moments as shown in Fig. 7 to illustrate its feasibility. Where Fig. 7(a,b), Fig. 7(d,e,g,h) are the deformed patterns in three different statuses respectively, and the Fig. 7(c,f,i) are the corresponding reconstructed objects. The real-time deformed patterns (Video S1) and reconstructed results (Video S2) are included in the Supplementary Materials.

## Conclusion

Compared with CGMP we proposed before, this proposed HCGMP efficiently avoids the filtering operation which may reduce accuracy on the whole in background components elimination. Especially when the spectrum of the captured deformed pattern is severely aliased, the proposed HCGMP can also work to its advantages. The remarkable advantage of this method is that both the background component and high frequency component of the original patterns can be well eliminated. Therefore, the proposed HCGMP holds the outstanding property of restraining noises. Moreover, compared with 4-step PMP, HCGMP requires less frame patterns and brings the almost equivalent results. Furthermore, the real-time measuring feature of HCGMP is still maintained though the single-shot feature is substituted by the two-frame feature. We believe it is a promising method which also has the potential to measure some deformations caused by a temperature change, a mechanical stress or other reasons. It deserves to be dug deeper.

## Supplementary information

Supplementary Information

Video S1

Video S2
