# Supplementary material for: High precision computer-generated moiré profilometry
Source: Sci Rep. 2019 May 24;9:7804. doi: 10.1038/s41598-019-44186-3 (PMC6534590; doi:10.1038/s41598-019-44186-3)
Supplement: Supplementary file 1 — Supplementary Information [file 41598_2019_44186_MOESM1_ESM.doc]

High precision computer-generated moiré profilometry

**Chengmeng Li, Yiping Cao,* Lu Wang, Yingying Wan, Guangkai Fu, Yapin Wang, Cheng Chen**

*Department of Opto-Electronics, Sichuan University,* *Chengdu, 610064, China*

[**ypcao@scu.edu.cn*](mailto:*ypcao@scu.edu.cn)

**Video S1 Real-time captured deformed pattern.** The acquisition rate is 80 fps.

**Video S2 Real-time reconstructed object.** The reconstruction rate is 40 fps.
